# Feasibility of an oral health promotion program among older people in geriatric care facilities, Shanghai, China: a pre/post-implementation study

**DOI:** 10.1186/s12877-024-04870-0

**Published:** 2024-03-19

**Authors:** Liyan Gu, Jingwen Zhang, Wenyao Chen, Yanqiu Weng, Lan Chen, Lingjuan Zhang

**Affiliations:** 1 Department of Neurology, NO. 905 Hospital of PLA Navy, Naval Medical University, 1328 Huashan Road, 200052 Shanghai, China; 2 Key Laboratory of Ministry of Education for Geriatric Long-term Care, Shanghai, China; 3 Education and Scientific Research Department of Clinical Nursing, Changhai Hospital affiliated to Naval Medical University, 168 Changhai Road, Shanghai, 200433 China; 4 Nursing Department, Shanghai General Hospital, Shanghai Jiao Tong University School of Medicine, Shanghai, 200080 China; 5 Shanghai Quality Control Center of Geriatric Care, 168 Changhai Road, Shanghai, 200043 China

**Keywords:** Oral health, Geriatric care, Older people, Feasibility, OHAT, Promotional program

## Abstract

**Background:**

The oral health of older people is closely related to their overall health. Timely and effective intervention in oral issues is necessary to maintain their overall health. This study aimed to evaluate the feasibility and effectiveness of an Oral Health Promotion Program (OHPP) in Geriatric Care Facilities (GCFs).

**Methods:**

The OHPP was implemented in two GCFs and evaluated using a pre/post-design. Questionnaires on self-efficacy and attitude for providing oral care were sent to 42 nurse participants before and three months after the implementation of the OHPP. Outcomes of 295 patient participants were assessed at four time points (T1-baseline, T2-one month, T3-two months, and T4-three months post-implementation) including Activities of Daily Living (ADL), Mini-Mental State Examination (MMSE), and Oral Health Assessment Tool (OHAT).

**Results:**

The oral health and daily activity ability of patient participants showed an improving trend at four time points pre/post-implementation of the OHPP. The proportion of patients with healthy mouths (OHAT: 0–3 points) increased from 29.8 to 67.8% and their scores of OHAT and ADL were significantly better at T4 compared to T1, T2, and T3 (*p* < 0.001). Self-efficacy (SE-PMC: T1 = 18.93 ± 3.18, T4 = 28.83 ± 6.56, *p* < 0.001) and attitude (A-PMC: T1 = 18.78 ± 3.09, T4 = 28.20 ± 6.03, *p* < 0.001) for oral care among nurse participants improved after the implementation of the OHPP.

**Conclusions:**

This study highlights the feasibility of implementing OHPP within GCFs, potentially enhancing the oral health and daily living activities of older individuals. Integrating the OHPP into routine care in geriatric settings is not only practical but also widely acceptable, offering a proactive approach to address oral health disparities among older residents. Stakeholders can maximize the impact of the OHPP by fostering collaboration among healthcare professionals, administrators, and residents, ultimately improving oral health outcomes and overall quality of life of older residents.

**Trial registration:**

ChiCTR2000035236 (registration date: 04/08/2020).

**Supplementary Information:**

The online version contains supplementary material available at 10.1186/s12877-024-04870-0.

## Introduction

The aging population is one of the most significant social transformations of the twenty-first century [1], and China now has the largest elderly population in the world. According to data released by the National Bureau of Statistics at the end of 2022, the number of people aged 60 and above in China exceeded 280 million, accounting for 19.80% of the country’s total population [2]. Oral health status has a direct impact on the quality of life of older people [3, 4]. As defined by the World Health Organization (WHO) [5], oral health is essential for maintaining overall health and quality of life and includes factors such as the absence of mouth or facial pain, oral infections or ulcers, and healthy teeth and gums with no decay, bleeding, or discoloration.

Oral health problems have emerged as a significant health concern for older people, posing threats to both their physical and mental well-being. In recent years, it has become recognized as a new geriatric syndrome [6, 7]. As the oral cavity serves as the entry point for the digestive system, tooth loss and dental caries can lead to difficulties with chewing and swallowing, resulting in reduced oral intake and malnutrition [8, 9]. Research indicates that professional oral care can decrease the likelihood of aspiration pneumonia, a potentially serious complication [10]. Additionally, studies have linked poor oral health with systemic diseases such as diabetes, hypertension, and dementia [11–14].

In 2019, China launched the Healthy Mouth Action Program (2019–2025) to promote oral health, particularly among older people. The program emphasizes the importance of understanding the link between oral health and overall well-being and encourages initiatives to raise awareness of oral health [15]. However, the fourth oral health epidemiological survey revealed that severe periodontitis affects 43.5% of older people aged 65–74, with a mean of 6.96 missing teeth [16]. It is conceivable that the oral health situation of older people in long-term care facilities is even worse which has been confirmed in previous studies [17, 18]. older people in long-term care facilities are at even greater risk of oral diseases due to factors such as limited self-care, co-morbidities, multiple medications, poor immune function, and infrequent oral health practices [18, 19]. Common oral health issues in this population include missing teeth, reduced periodontal support, reduced saliva production, periodontitis, and low oral hygiene levels [20, 21]. Lack of oral health awareness is one of the main causes of patients’ poor oral health condition in geriatric care facilities (GCFs). Research indicates that only 47.6% of older people have adequate oral health knowledge, and 42.1% brush their teeth less than twice daily, while 27.7% do not brush their teeth correctly, and only 0.8% floss regularly [22].

Previous literature suggests that scientific and standardized oral care is a crucial aspect of improving oral hygiene in older people, as it can reduce infection risk, restore oral function, promote cleanliness, decrease complications, enhance comfort, and raise awareness of oral health care [23]. Evidence-based programs for older people’s oral care have been successfully implemented in western countries, such as New York State nursing homes, where part-time dental hygienists were utilized to coach staff and improve oral care quality [24]. Similarly, Red et al. [25] established an evidence-based oral care program for older people in long-term care facilities, which led to significant improvements in patients’ oral health outcomes and caregivers’ knowledge. However, China lacks uniform oral assessment tools and training for healthcare professionals, and traditional methods such as oral wiping are still prevalent [26]. Despite this, Chinese nursing scholars, including Cao Junyan [27] and Bo Lin [28], have developed evidence-based protocols and standardized programs for oral care among older people with cognitive impairment and swallowing problems, respectively, which have led to improved quality of care.

To the best of our knowledge, most studies on oral health in Chinese older people of GCFs have been limited to epidemiological surveys, and there have been few reports on multicenter projects promoting the translation of oral care-related evidence. Given the complexity of physical conditions among GCF residents, it is challenging to improve oral hygiene using a single approach. Previously, the research team investigated the oral health status of GCF residents [29] and developed an instrument to measure nurses’ self-efficacy and attitudes towards oral care [30]. Building on this foundation, an evidence-based oral health promotion program (OHPP) was developed for institutionalized older people and provided standardized oral care training and guidance to nursing professionals in GCFs. This study aims to examine the feasibility of the OHPP in GCFs and evaluate its effectiveness.

## Methods

### Study design

Between August 4 and October 31, 2020, a feasibility study was conducted in two GCFs using pre/post-assessments on patients and nurses. Feasibility studies focus on the question of “can it work?” when testing the acceptability and applicability of an intervention for a particular group [31]. To obtain meaningful inferences from the data within the research timeframe, an expert panel suggested a sample size of 30 nurse participants and 200 patient participants, which was considered practical. The ethics committee of Shanghai General Hospital affiliated with Shanghai Jiao Tong University School of Medicine approved the study under the approval number of 2020KY040. Additionally, the study was registered in the Chinese Clinical Trial Registry under the registration number ChiCTR2000035236 (date: 04/08/2020). The full trial protocol could be accessed at https://www.chictr.org.cn/showproj.html?proj=54149.

### Participants

**Fig. 1:**
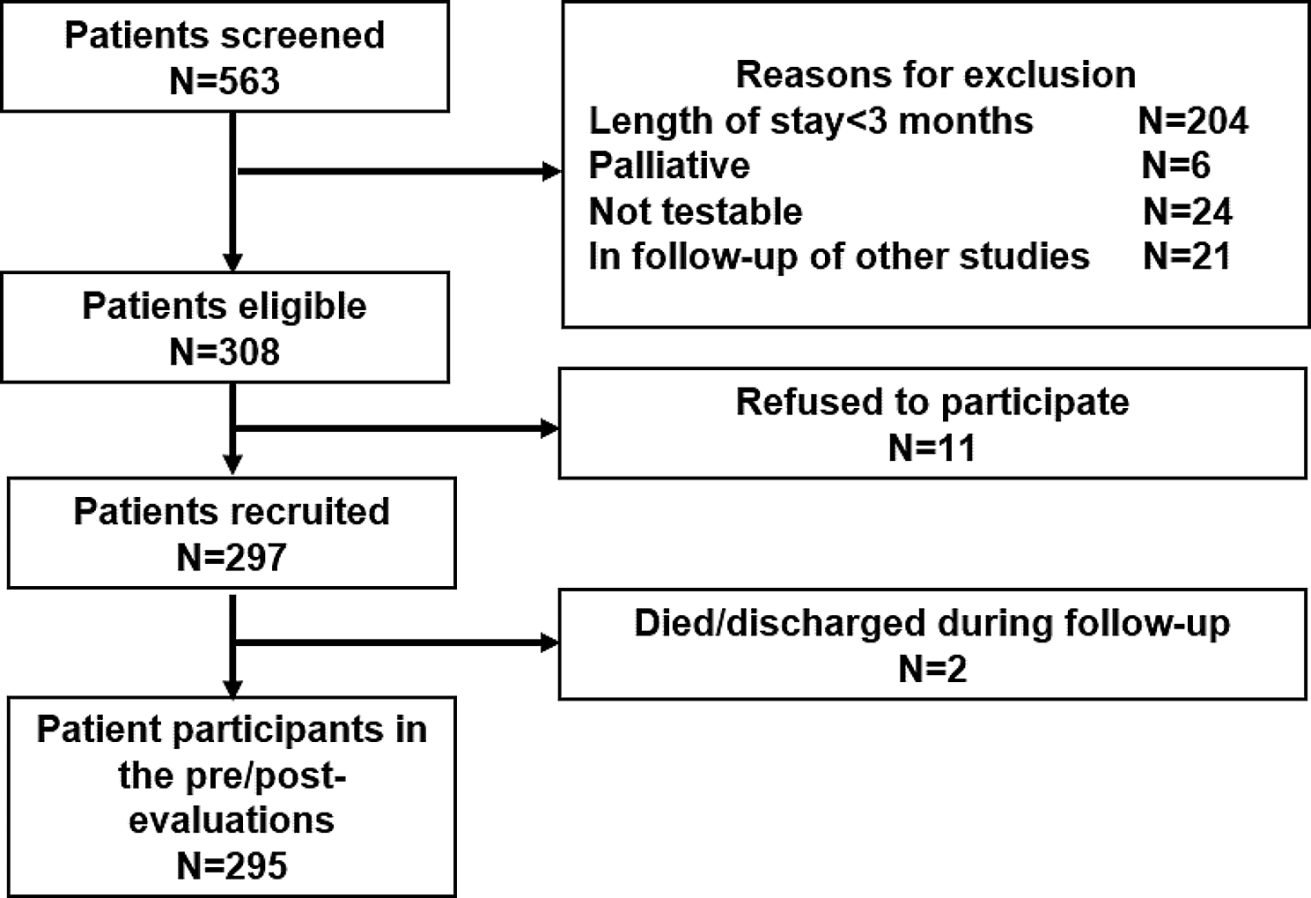
Patient participants flow of the study

Table 1 outlines the inclusion and exclusion criteria for patient and nurse participants. Convenience sampling was used to recruit participants from two 300-bed GCFs that provide treatment and care for older people with chronic diseases, many of whom have complex care needs and deteriorating functions. Forty-two nurses were invited to participate in the study after providing informed consent and receiving permission from their ward directors, nursing managers, and institution administrators. The research team trained the nurse participants to implement the OHPP. Potential patient participants (out of a population of 563 residents) were screened by both nurse participants and researchers using the inclusion/exclusion criteria outlined in Table 1, and 295 of them participated in the pre/post-implementation study. Nurse participants made initial contact with patients or their caregivers/guardians, after which researchers obtained formal informed consent. Figure 1 illustrates the flow of patient participation in the study.

**Table 1:** Participant eligibility criteria

	Inclusion criteria	Exclusion criteria	withdraw criteria
Nurse participants	officially Registered Nurses in GCFs; nursing experience in GCFs over 1 year; informed consent and voluntary participation	off-site training or sick leave	
Patient participants	over 60 years old; stayed in GCFs at least three months; informed consent from patients or their caregivers/guardians	having any life-threatening, serious medical problems; inability to undergo oral assessment or cooperate; in follow-up of other studies	death during the study; discharged or transferred to another institution and unable to continue with the intervention; patients or their legal guardians requesting to withdraw from the study and not completing the full intervention and data collection

### The OHPP intervention

The OHPP was developed using Delphi and evidence-based methods, following the Template for Intervention Description and Replication (TIDieR) guidelines [32]. The aim of OHPP was to establish a standardized procedure for oral care of older people in GCFs, with nurses as providers. The intervention’s target, content, frequency, and effectiveness evaluation were included in the OHPP plan sheet and record sheet.

The research team and nursing administrators of the two GCFs collaborated to discuss the study’s objectives, importance, implementation, assessment, and relevant requirements, building mutual understanding, trust, and support. Nurse participants received initial training on the key points and quality control of the OHPP intervention, including knowledge and skills training. They assessed oral health status of patients, created care plans, and maintained records throughout the intervention. Nurse participants provided weekly feedback on any special situations such as interruptions or lack of cooperation. The research team maintained close contact and were prepared to address any queries to ensure quality control and supervision of the OHPP implementation.

### Measurements

A set of validated assessment tools were selected to evaluate the outcomes of patient participants and the self-efficacy and attitudes towards oral care of nurse participants. These assessments include the Activities of Daily Living (ADL), Mini-mental State Examination (MMSE), Oral Health Assessment Tool (OHAT), Self-efficacy for Providing Mouth Care (SE-PMC) and Attitudes for Providing Mouth Care (A-PMC) Scale. The details of these assessments are summarized in supplement files.

The OHAT is a practical tool for measuring overall oral health which was verified by Chalmers et al. [33]. It has been translated into many versions and used widely [34, 35]. Items of the OHAT composed of lips, tongue, gums and tissues, saliva, real teeth, dentures, oral cleanliness, and dental pain. This instrument could be applied to patients with cognitive impairment because it does not require patients to express themselves. Each item receives a score between 0 (healthy) and 2. (unhealthy). Three categories are possible based on the overall score, which runs from 0 to 16: “healthy mouth” (0–3), “changing mouth” (4–8), and “unhealthy mouth” (9–16). With a Cronbach’s value of 0.710 and test-retest reliability of 0.811, the Chinese version of the OHAT can be used to evaluate the oral health of Chinese older people [36].

The SE-PMC and A-PMC was used to assess the self-efficacy and attitude of nursing staff who provide oral care. They were developed by Wretman [37] and have been applied and tested in geriatric nursing institutions. In our previous work, the Chinese version of SE-PMC and A-PMC was translated, culturally adjusted, and psychometrically tested [30]. The Chinese version of SE-PMC (11 items, 3 dimensions) and A-PMC (11 items, 2 dimensions) included 22 items and were rated on a Likert 4-point scale ranging from “1-strongly disagree” to “4-strongly agree” (maximum score = 88; minimum score = 22). Higher scores indicate nurses’ better self-efficacy and attitudes in delivering dental care. The Cronbach’s coefficient of the Chinese Version of SE-PMC and A-PMC was 0.831 and 0.768. In addition, test-retest reliability was 0.809 for SE-PMC and 0.811 for A-PMC.

### Data collection

Pre- and post-evaluations of the OHPP implementation were conducted in two 300-bed GCFs, one private and one public. Baseline data collection was conducted to record the initial characteristics and status of the participants prior to the implementation of OHPP. The patient participants’ data was collected by trained nurse participants who followed standardized courses for assessment criteria, recording methods, clinical implementation, and documentation of the OHPP intervention. Patient socio-demographic information, such as age, gender, payment, diet method, frequency of family visits, bacterial plaque, and diagnosis, was extracted from medical records. Nurse participants also gathered patient data on ADL, MMSE, and OHAT at four time points (T1-baseline, T2-one month, T3-two months, and T4-three months post-implementation), along with their oral health behaviors such as brushing, rinsing, and oral care procedures via interviews and observations. Additionally, the SE-PMC and A-PMC of nurse participants was evaluated at baseline and three months after OHPP implementation to improve clinical supervision and ensure the delivery of OHPP interventions. Nurse administrators was educated to enhance clinical supervision. Furthermore, rigorous research data management was employed through double-checks, random checks, and proofreading to assure data validity and correctness.

### Data analysis

The study utilized SPSS 23.0 software for statistical analysis and GraphPad Prism 8 to visualize the statistical results. Continuous data were presented as means ± standard deviations and median, while categorical variables were reported as percentages or frequencies. As the variables were not normally distributed, Friedman analysis was used to compare OHAT, ADL, and MMSE scores at the four time points. Chi-square testing was employed to compare the grading distribution of OHAT and its items at T1 and T4. Paired t-tests were used to compare SE-PMC and A-PMC scores (continuous variables) at T1 and T4. A significance level of *P*<0.05 was used to determine statistical significance.

## Results

### Demographic characteristics of the participants

Table 2 presents the characteristics of the study participants, including their age, gender, ADL score, diagnosis, and baseline oral health assessment. The patient population had a mean age of 83.69 ± 9.82 years, with 192 (65.1%) females and 103 (34.9%) males. The median ADL score was 10, with the 25th and 75th percentiles being 0 and 35, respectively. Most patients (*n* = 258, 87.5%) were diagnosed with neurological diseases. Baseline oral health assessments revealed that 73 (24.7%) patients had no visible plaque, 98 (33.2%) had probe-accessible plaque, 107 (36.3%) had moderate plaque, and 17 (5.8%) had severe plaque.

The nurse participants in this study had an average age of 31.42 ± 5.83 years and an average length of employment of 10.46 ± 6.01 years. Of the participants, 39 (95.1%) were female and 2 (4.9%) were male. Additional information was displayed in Table 3.

**Table 2:** Socio-demographic characteristics of the patient participants in the GCFs (*n* = 295)

Variables	Categories	(Mean ± SD)/ [*n* (%)]/ [Median (P25, P75)]
Age		83.69 ± 9.82
Gender	Male	103 (34.9)
	Female	192 (65.1)
Payment	Medical insurance	290 (98.3)
	Self-paid	5 (1.7)
Diet method	Normal feeding	207 (70.2)
	Nasogastric tube	88 (29.8)
Bacterial plaque	None	73 (24.7)
	Probe-accessible	98 (33.2)
	Moderate	107 (36.3)
	Severe	17 (5.8)
Dentures	Yes	22 (7.5)
	No	273 (92.5)
Frequency of family visits	< 1 time per week	93 (31.5)
	1–3 times per week	157 (53.2)
	> 3 times per week	45 (15.3)
Diagnosis	Neurological diseases	258 (87.5)
	Respiratory diseases	35 (11.9)
	Digestive diseases	2 (0.7)
ADL		10 (0, 35)

**Table 3:** Socio-demographic characteristics of nurses (*n* = 42)

Variables	Categories	(Mean ± SD)/ [*n* (%)]
Age		31.42 ± 5.83
Gender	Male	2 (4.9)
	Female	39 (95.1)
Education	Junior College	6 (14.6)
	Bachelor’s degree or above	35 (85.4)
Professional title	Junior nurse	10 (24.4)
	Senior nurse	17 (41.5)
	Supervisor nurse or above	14 (34.1)
Length of employment (year)		10.46 ± 6.01

### Pre/post-evaluations of patient participants’ OHAT, ADL and MMSE

**Fig. 2:**
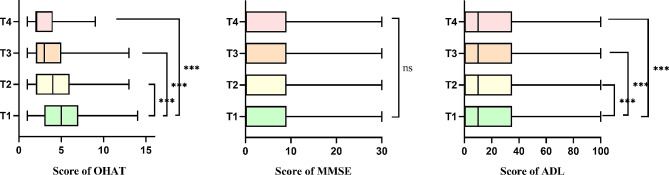
Comparison of patient participants’ OHAT, MMSE and ADL at T1, T2, T3 and T4 (*n* = 295) using Friedman analysis. T1, baseline; T2, one month post-implementation; T3, two months post-implementation; T4, three months post-implementation; ns, not significant; ***, *P* < 0.001

**Fig. 3:**
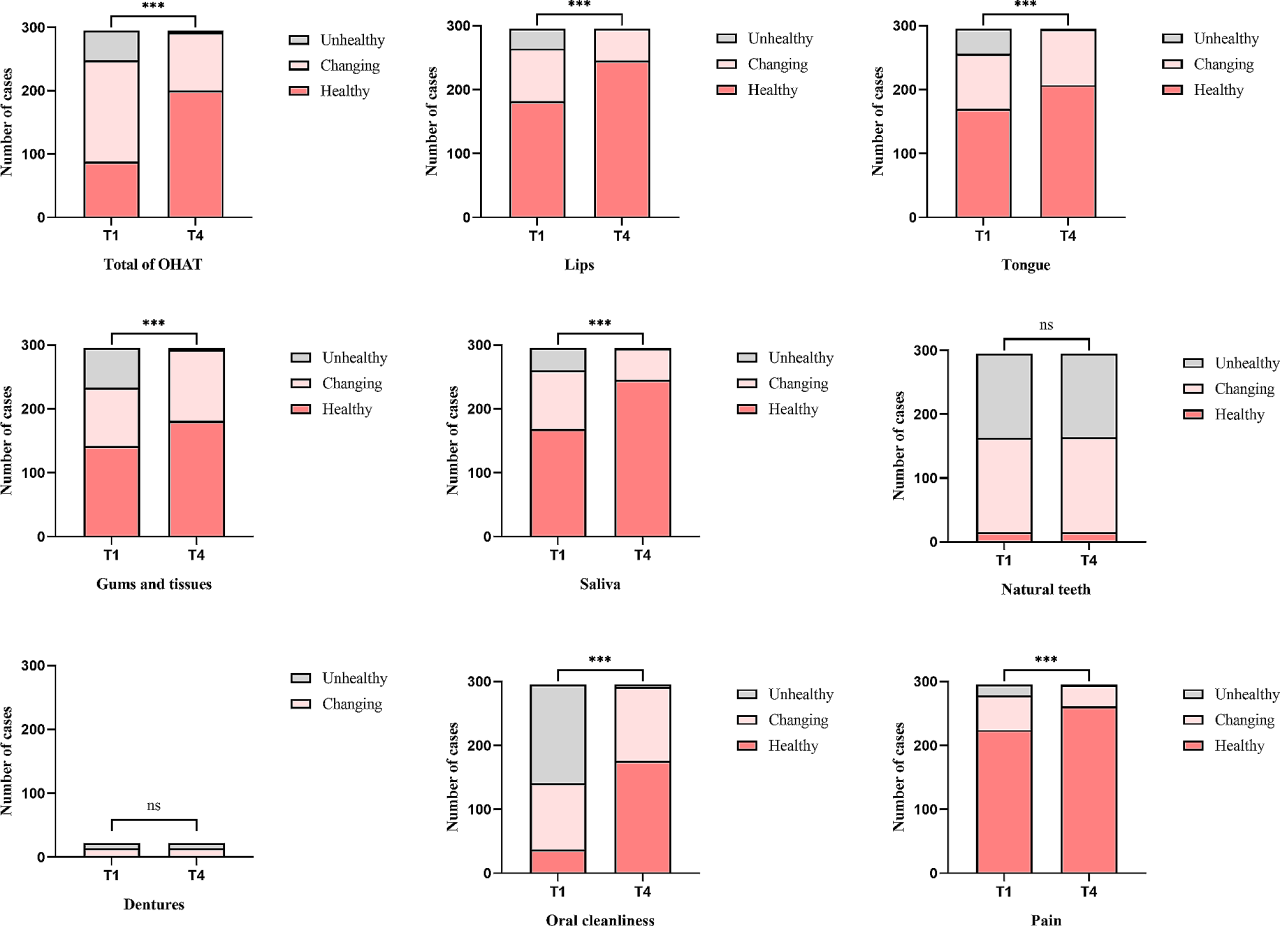
Grading distribution of patient participants’ OHAT and its items (*n* = 295) using Chi-square testing. Healthy (OHAT: 0–3 or item score = 1): maintained by usual care only; Changing (OHAT: 4–8 or item score = 2): observation and monitoring are required; Unhealthy (OHAT > 9 or item score = 3): care required and professional dental appointments should be arranged. T1, baseline; T4, three months post-implementation; ***, *P* < 0.001

Figure 2 presents the OHAT, ADL, and MMSE scores of patient participants at four time points: baseline (T1), one month post-implementation (T2), two months post-implementation (T3), and three months post-implementation (T4). From T1 to T4, the medians for OHAT were 5, 4, 3 and 2, respectively, while those for MMSE and ADL were consistently 0 and 10. Patient assessments for OHAT and ADL were significantly better at T4 compared to T1, T2, and T3 from results of Friedman analysis (*P* < 0.001). Figure 3 compares the grading distribution of OHAT and its items before and after implementation of OHPP. At T1, patients with changing mouth had the highest proportion (*n* = 160, 54.2%), followed by healthy mouth (*n* = 88, 29.8%) and unhealthy mouth (*n* = 47, 15.9%). After three months of intervention (T4), the proportion of healthy mouth increased to 67.8% (*n* = 200), while changing mouth and unhealthy mouth decreased to 31.2% (*n* = 92) and 1% (*n* = 3), respectively. The change in the proportion of OHAT assessment grading before and after the intervention was statistically significant. Significant differences were also observed in items such as lips, tongue, gums and tissues, saliva, oral cleanliness, and pain, except for natural teeth and dentures when comparing the rating of each item of the OHAT before and after the intervention.

### Pre/post-evaluations of nurse participants’ SE-PMC and A-PMC

**Fig. 4:**
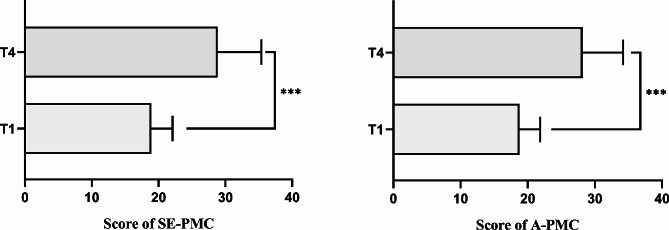
Comparison of SE-PMC and A-PMC of nurse participants pre and post implementation (*n* = 41) using paired t-tests. T1, baseline; T4, three months post-implementation; ***, *P* < 0.001

Figure 4 displays the pre/post-evaluations of nurse participants’ SE-PMC and A-PMC. The average score of SE-PMC was 18.93 ± 3.18 at T1 and 28.83 ± 6.56 at T4, while that of A-PMC was 18.78 ± 3.09 at T1 and 28.20 ± 6.03 at T4. The scores of the SE-PMC, A-PMC and their sum at T4 were significantly higher than those at T1 (*P*<0.001).

## Discussion

This study investigated the feasibility and acceptability of integrating OHPP into routine geriatric care settings. Nurse participants underwent training on OHPP during the pre-intervention phase. Subsequently, a standardized implementation of OHPP interventions was conducted on patient participants in two GCFs over a period of three months. Overall, these findings underscore the viability of implementing OHPP in geriatric care settings, indicating its potential to positively impact the oral health outcomes of older individuals residing in GCFs.

To comprehensively assess the feasibility and effects of OHPP, a dynamic perspective was adopted, analyzing outcomes at both patient and nurse levels. Oral health and daily activity ability of patient participants were evaluated at four time points: baseline, one month, two months, and three months post-implementation. The analysis revealed a notable improvement trend, with the median scores of OHAT decreasing from five to two. Specifically, the proportion of patients with a healthy mouth (OHAT: 0–3 points) increased significantly from 29.8 to 67.8%, while the percentage of those with changing and unhealthy oral health decreased from 54.2 to 31.2% and 15.9–1.0%, respectively. Moreover, the study observed positive changes in nurse participants’ self-efficacy (SE-PMC) and attitude (A-PMC) towards oral care following the implementation of OHPP. These improvements were statistically significant compared to baseline data.

The implementation of OHPP has led to improvements in oral care quality at the geriatric care settings. From a management perspective, the dissemination and implementation of evidence-based interventions in clinical practice involve multiple levels (organizational, practitioner, and patient), representing a top-down or bottom-up change process to promote practitioner behavior change [38]. The implementation of OHPP in two GCFs has also facilitated nursing administration, with a series of implementation strategies embedded in clinical contexts such as establishing implementation teams, ongoing training, and quality control being key drivers of OHPP adoption in these settings. In this study, patient participants were categorized into different groups based on their baseline oral conditions, including tooth brushing, oral wiping, denture care, and dementia care groups. Each patient was given an individualized oral care plan and a record sheet to be included in their personal medical history. The responsible nurse was required to ensure that specific requirements of the plan were completed or supervised on a daily basis. Any exceptional circumstances preventing completion were recorded in the notes. Additionally, there was a weekly “reflection log” for nursing staff to reflect on their oral care work, address problems, and improve their care practices moving forward.

According to the results, the implementation of OHPP effectively improved several health outcomes for older people, including OHAT and ADL. OHAT was the main health outcome measure in this study, reflecting the effectiveness of the OHPP intervention. The results showed that OHAT scores were higher at T1 than that at T2, T3, and T4, with significant differences (*P* < 0.001), indicating an improvement in oral health over time for older people. Researchers screened the oral health of older people who had been hospitalized for more than three months in GCFs and monitored their OHPP status at different time points within three months of implementation. The improvement in oral health at T4 was more significant than at T2 and T3, indicating the need for sustained implementation of OHPP. Due to barriers at individual and organizational levels [39] (e.g. lack of oral care equipment, absence of guidelines, shortage of staff, time constraints, inadequate knowledge, poor supervision, high workload, and not being a priority), healthcare professionals might be hindered from performing standard and effective oral care. These barriers should arouse attention from stakeholders to avoid older people from missing the best opportunity for oral health interventions. Additionally, although the median ADL of T1-T4 did not change, Friedman analysis showed that older people’s ADL also gradually improved, possibly because OHPP encouraged self-care measures such as brushing teeth and mouthwash for those with remaining self-care abilities, consistent with Shiraisi’s [40] research. Nevertheless, the MMSE of older people did not change throughout T1 to T4. This may be due to the continuous decline in cognitive function of older people [41], which is difficult to reverse or improve through oral care measures.

Comparing grading distribution of patient participants’ OHAT and its items at T1-T4, grading of “natural teeth” and “dentures” did not significantly improve either before or after the intervention. The assessment found that the majority of older people had a score of 2 (unhealthy) for the “natural teeth” item based on the number of natural teeth remained. The loss of natural teeth is an irreversible process. Similar to the scoring of “natural teeth” assessment, the assessment on dentures found that 33 patients who wore dentures had worn theirs for a longer period of time, with partial loss or breaks, whereas 262 respondents who did not use dentures had missing teeth. This might be explained by the fact that denture replacement is a tough task for older people concerning medical accessibility and thus necessitates professional dental care as supported by Yuka’s research [42]. Other items, including “lips”, “tongue,” “gums and tissues,” “saliva,” “oral cleanliness,” and “pain,” showed significant improvements before and after the intervention. Overall, the implementation of OHPP has shown positive effects on health outcomes in older people, highlighting the importance of sustained implementation and early intervention. However, contrasting findings in the literature suggest a potential decline in effectiveness when oral health interventions extend beyond a three-month duration [43]. This disparity in outcomes could be attributed to various factors that merit careful consideration. One plausible explanation is the diminishing adherence and sustainability of oral health practices over an extended intervention period. It is conceivable that participants may find it challenging to maintain the same level of engagement and compliance with oral health recommendations over an extended timeframe. This phenomenon might be influenced by factors such as motivation, habit formation, and competing priorities. Furthermore, the nature of the oral health intervention itself may play a role. The initial enthusiasm and responsiveness of participants may wane over time, leading to a gradual decline in the intervention’s impact. Additionally, the intervention content and delivery methods may need to be adapted to maintain participant interest and participation over an extended duration. For future research, it is essential to delve deeper into the mechanisms influencing the temporal dynamics of oral health interventions. Longitudinal studies with varied intervention durations and comprehensive assessments of participant adherence, motivation, and intervention fidelity can provide valuable insights.

In this study, nurse participants’ SE-PMC and A-PMC rating increased significantly at post-implementation evaluation. It thus might be inferred that the OHPP contributed to the improvement of nurse participants self-efficacy and attitude toward oral care and might be endorsed by more nursing professionals. The reason for this result could be that the OHPP prompted nurses to think proactively and improved their problem-solving skills. As reported by literature [44], self-efficacy is an individual’s judgment of whether a task can be accomplished and the psychological activities and behaviors adopted to achieve desired outcomes. The OHPP intervention has established a standardized oral health assessment mechanism led by nurses. Trained nurses with sound self-efficacy and attitude toward oral care could complete oral assessments and maintain contact with dental professionals to reduce barriers to oral care for residents in GCFs.

For further implications, a comprehensive approach was proposed that involves the utilization of OHAT as a standard practice for evaluating the oral health status of newly admitted patients. This assessment will serve as a foundation for tailoring the OHPP to meet the specific oral health needs of each patient, ensuring a patient-centered approach to oral health care. To effectively promote the OHPP and ensure its sustained adoption, the following strategies were recommended: **(a) Training and Education**: We advocate for the development and implementation of routine training opportunities targeted at nurses and caregivers. This could include a range of educational formats such as brief lectures, video tutorials, and expert-led group sessions, designed to enhance the direct caregivers’ knowledge and skills in oral health care; **(b) Collaboration with Dental Professionals**: Maintaining ongoing communication and collaboration with dental professionals is essential. This collaboration will help in reducing barriers to dental treatment for patients by facilitating timely referrals, sharing of best practices, and co-management of patients with complex oral health needs; **(c) Administrative Support**: The role of nursing administrators and educators is pivotal in promoting the OHPP. Administrative support be manifested through the allocation of resources for training programs, recognition of oral health care as a priority area in patient care, and the integration of the OHPP into the routine care protocols within the GCFs. This will not only institutionalize the program but also ensure its sustainability.

### Limitations

Despite our efforts to ensure the scientific rigor of this study, its several limitations should be acknowledged. First, this study only included pre/post-implementation data of the participants, lacking a multi-center randomized controlled trial (RCT) to further confirm the effectiveness of the Older Health Promotion Program (OHPP). The limitation of this design is that it may not fully capture the long-term effects and potential variability of the OHPP intervention. Future studies should consider using a multi-center RCT design to improve the external validity and generalizability of the results. Second, this implementation study was conducted in two 300-bed Geriatric Care Facilities (GCFs) with better infrastructure and human resources, which may have biased the results. This choice was based on the availability of facilities and human resources but limits ability to generalize the study findings to GCFs with less infrastructure and human resources. Future research should consider involving GCFs of varying sizes and conditions to ensure the universality of the results. Third, a mixed-methods design was desired to gain a more comprehensive understanding of the acceptability of OHPP among older adults and nursing professionals. The current study focused on quantitative data, which may not fully capture participants’ feelings and experiences towards OHPP. Using qualitative methods can provide deeper insights, helping to understand the acceptability of the intervention and its variation across different groups. Future studies should combine quantitative and qualitative data for a more comprehensive understanding.

To overcome these limitations, future researchers should consider the following to overcome these limitations: employing a multi-center RCT design, expanding the study to include GCFs under different conditions, and using a mixed-methods design to enrich understanding of intervention acceptability. Through these approaches, future research can provide more convincing evidence to support the effectiveness and applicability of OHPP in the elderly population.

## Conclusions

In this study, the feasibility of the OHPP intervention was examined in geriatric care settings and shown that it was viable among both patients and health care providers. Moreover, this study provides a scientific and systematic methodological reference for the application of clinical practice protocols in geriatric care for oral health promotion. The OHPP intervention deserves further testing in a cluster randomized trial to determine whether its implementation would lead to better outcomes for patients and health care providers.

### Electronic supplementary material

Below is the link to the electronic supplementary material.

Supplementary Material 1

Supplementary Material 2

## Data Availability

The datasets used and analyzed in the current study are available from the corresponding author on reasonable request.

## Abbreviations

ADL: Activities of Daily Living
A-PMC: Attitudes for Providing Mouth Care
GCF: Geriatric Care Facilities
MMSE: Mini-Mental State Examination
OHPP: Oral Health Promotion Program
OHAT: Oral Health Assessment Tool
SE-PMC: Self-efficacy for Providing Mouth Care
TIDieR: Template for Intervention Description and Replication
WHO: World Health Organization
