# Supplementary material for: Feasibility of an oral health promotion program among older people in geriatric care facilities, Shanghai, China: a pre/post-implementation study
Source: BMC Geriatr. 2024 Mar 19;24:272. doi: 10.1186/s12877-024-04870-0 (PMC10953076; doi:10.1186/s12877-024-04870-0)
Supplement: Supplementary file 1 — Supplementary Material 1 [file 12877_2024_4870_MOESM1_ESM.docx]

**Oral Health Promotion Program (OHPP) for older people in geriatric care facilities**

| **Categories** | **Entry Content** | **Evidence Level/**  **Recommendation Strength** |
| --- | --- | --- |
| Oral Assessment | 1.Nursing staff assess the oral health status of older people immediately during their admission to a geriatric care facilities. | 1a / Grade A |
|  | 2.Actively negotiate human resources, cooperate with each other, and regularly evaluate the oral health status of older people at appropriate time intervals. If necessary, ask caregivers to assist in completing the assessment. | 1a / Grade A |
|  | 3.Nursing staff regularly evaluate the oral health status of patients with dementia. | 1a / Grade A |
|  | 4.Nursing staff regularly check the oral health status of patients with tube feeding. | 5a / Grade A |
|  | 5.Nursing staff conduct periodontal assessment on older people with signs or symptoms of gum disease. | 5a / Grade B |
|  | 6.OHAT, an oral health assessment tool, is used for the assessment. | 5a / Grade A |
|  | 7.As part of the admission assessment, nursing staff should obtain and record the patients’: (i) oral health history, including the patient's self-perceived oral health status, preferences (whether or not they have a sweet tooth, midnight snack, etc.), and any previous oral treatment (e.g., toothwash, put in a false tooth, surgery, etc.); (ii) chewing performance; (iv) oral self-care ability. | 3c / Grade A |
| **Education & Training** | 8.Geriatric care facilities provide oral care education and training to nursing staff and caregivers with the assistance of dental professionals. The training is conducted through PPT lectures, distribution of learning materials, practical training, and WeChat push, etc. An assessment system is also established to strengthen the nursing staff's attention to oral care. Specific contents include:  (i) oral-related theories and knowledge, including: the current status of oral health of the elderly in China, the definition of oral health, the importance of oral care, risk factors of oral diseases and prevention methods, etc;  (ii) practical oral care skills, including toothbrush and denture cleaning techniques;  (iii) interactive practical training: operation instruction of oral care for older people, nurses who receive training respond to the demonstration, and the teacher corrects errors and provides guidance. | 5a / Grade A |
|  | 9. Geriatric care facilities send nursing staff to attend lectures and training on evidence-based nursing to receive systematic and rigorous learning. | 5a / Grade A |
|  | 10. Geriatric care facilities hold regular meetings to discuss the problems encountered during the implementation of the program. | 5a / Grade A |
| **Draw Up a Plan** | 11. Oral care programs are developed with the participation and collaboration of interprofessional team members. | 5a / Grade A |
|  | 12. Nursing staff are aware of the ways to seek guidance and advice on oral care or use of help guides. | 5a / Grade A |
|  | 13. It is recommended that nursing staff evaluate the environment and the needs and cooperation of older people before implementing nursing measures, and provide targeted nursing measures based on these factors. | 4a / Grade A |
|  | 14. Record the oral care needs of older people in the nursing record sheet, implement an interprofessional approach to providing oral care, allocate sufficient time for nursing staff, and clarify their responsibilities to overcome deficiencies in human resources and time allocation. | 4b / Grade A |
| **Nursing Implementation** | 15. Nursing staff should follow a comprehensive and integrated oral care program to provide routine or specialized care and treatment for institutional patients. Specific contents include:  (i) personalized oral care program;  (ii) step-by-step instructions for oral care, including brushing and denture brushing;  (iii) determine the required oral care tools and supplies;  (iv) assist patients with oral exercises, oral self-care and salivary gland massage to stimulate the salivary glands and increase the rate of oral saliva production through muscle movements of the head, neck, face and tongue. | 5a / Grade A |
|  | 16. Referrals can be made to in-house dental professionals or dental professionals in the community to diagnose and provide the required treatment based on the individual personalized needs. | 5a / Grade A |
|  | 17. For patients with natural teeth, it is recommended to use a soft toothbrush and fluoride toothpaste for brushing, as well as therapeutic fluoride products and therapeutic chlorhexidine gluconate products. Brush teeth at least twice a day for approximately 2 minutes each time. If brushing with a manual toothbrush is difficult, use a power or sonic toothbrush. | 5a / Grade A |
|  | 18. If possible, use the patients’ preferred over-the-counter product, such as mouthwash or toothpaste; if patients use sugar-free gum, consider gum containing xylitol. | 5a / Grade B |
|  | 19. Conduct professional oral cleaning care for older people once a week. | 5a / Grade A |
|  | 20.Older people with teeth should limit sugary, refined and processed foods, caries prone snacks, acidic foods, and carbonate beverages to protect natural teeth from caries damage and erosion problems. After consuming acidic foods and beverages, the mouth should be rinsed with plenty of water. | 5a / Grade B |
|  | 21. The oral mucosa of older people with edentulous jaws should be rinsed with plenty of water after meals to remove residual food particles, and cleaned with a soft toothbrush or gauze. | 5a / Grade A |
|  | 22. Unless otherwise recommended by a dental professional, dentures should be removed at night and stored in water with denture cleaning solution. Daily oral care (such as brushing teeth, removing food debris) should be provided to older people with full or partial dentures. Dentures should be brushed at least twice a day with a non-abrasive denture cleaner or liquid soap (do not use toothpaste). Mechanical cleaning should be combined with chemical cleaning agents, and denture brushes should be dried carefully. | 5a / Grade A |
|  | 23.Older people should be provided with containers to hold their dentures. Dentures, denture containers and all other oral care supplies (such as toothbrushes, toothpaste and lip balm) should be labeled with individual's name to ensure that correct infection control measures are followed and that supplies are not inadvertently shared among different patients. | 5a / Grade A |
| **Preventive Measures** | 24. Measures such as controlling and managing common risk factors such as smoking, diabetes, and limiting alcoholic beverages are important components of periodontal disease prevention, in which doctors can play an important role in this. Periodontal care should start as early as possible, not only to prevent tooth loss, but also to prevent the potential effects of underlying diseases in older people. | 5a / Grade B |
|  | 25. Regular dental checkups every 3-12 months. It is recommended that nursing staff make clinical judgment based on the individual patient needs and decide when to refer the patient to a dental professional for more comprehensive dental assessment and treatment. | 3a / Grade B |
|  | 26.Patients with severe tooth loss and denture wearers should receive appropriate dietary advice and food preparation counseling to improve nutritional intake. | 5a / Grade B |
|  | 27. Geriatric care facilities develop oral health education materials and provide support to patients to meet the needs of all patients, especially those with complex oral care needs. | 5a / Grade A |

**Implementation Group of OHPP**

| **Roles** | **Title** | **Main Responsibilities** |
| --- | --- | --- |
| Project leader | Chief superintendent nurse | Organization, planning, and guidance of the project |
| Dental professionals | Associate chief physician | Provide professional dental guidance and treatment, assist in answering difficult questions |
| Research group members | Associate chief senior nurse / supervisor nurse / nurse | Regular updates of evidence, continuous improvement of programs, data collection and analysis |
| Intervention group members | Supervisor nurse / nurse practitioner | Project actual operation, work records, data collection, and problem feedback |
| Quality control team members | Co-chief superintendent nurse / supervisor nurse | Monitor the quality of project operations, organize education and training, provide support and assistance |

**Oral Care Training Course Arrangement**

| **Course Content** | **Duration of training** |
| --- | --- |
| Basic theories and knowledge related to oral cavity | 45 min |
| Explanation on the causes, hazards, and prevention of oral diseases in the elderly | 60 min |
| Oral health assessment, use of oral care products | 60 min |
| Practical oral care skills, oral care procedures | 60 min |
| Situational simulation of oral care for elderly patients | 45 min |
| Nurses receiving training respond to the demonstration, and the instructor corrects errors and instructs | 60 min |
