# Supplementary material for: Feasibility of an oral health promotion program among older people in geriatric care facilities, Shanghai, China: a pre/post-implementation study
Source: BMC Geriatr. 2024 Mar 19;24:272. doi: 10.1186/s12877-024-04870-0 (PMC10953076; doi:10.1186/s12877-024-04870-0)
Supplement: Supplementary file 2 — Supplementary Material 2 [file 12877_2024_4870_MOESM2_ESM.docx]

# Oral Health Promotion Plan Sheet for the Older Persons in Geriatric Care Facilities (GCFs)

| Goals | Overall goal: The establishment of a standardized implementation system and solution for the oral health of older persons in GCFs.  Specific objectives: to improve the oral health of the older persons | |
| --- | --- | --- |
| Intervention targets | Older persons in GCFs | Bed number: Name: |
| Interventionists | Nurse or nurse's aide in a  geriatric care facility | Nurse: Nursing assistant: |
| Intervention  time | During the patient's  hospitalization | Start date: End date: |
| Intervention content | **A: Oral health assessment.** | |
|  | - Purpose of the assessment: To assess the oral health status of the older persons in GCFs - Assessment Tool: Oral Health Assessment Tool (OHAT) - Assessment requirements: complete assessment within 24 hours of patient admission; at least once a month; as often as needed - Assessment performed by: | |
|  | **B: Oral health behavioral interventions:** | |
|  | **Brush teeth** | **Oral Care** |
|  | □ Brush teeth independently  □Brush teeth with assistance | □Inability to eat by mouth: hyperthermia, fasting, nasal diet  □Cognitive impairment and inability to cooperate with toothbrushing (MMSE<24)  □Live dependently and inability to cooperate with toothbrushing (BI<40)  □Swallowing or chewing disorder or food residue retention   - Palliative status |
|  | - **Brushing tools.**   - Electric toothbrush   - Manual toothbrush - **Toothpaste selection.**   - Fluoride toothpaste - **Brushing frequency.**   - After waking up in the morning 8 AM   - Before bedtime 8 PM - **Brushing duration.**   □≥3min/time | - **Oral care tools.**   □Repeated sterilization oral care package (with 16 cotton balls)  □Disposable oral care kit (with 16 cotton balls)  □Modified oral care kit (with 1 toothbrush)   - **Oral care solutions.**   - 0.9% saline   - Compound Chlorhexidine mouthwash   - Other mouthwashes: |

|  |  | - **Frequency of oral care.**   □ 8 AM in the morning □ 2 PM in the afternoon □ Other.   - **Duration of oral care:** □≥5min/time - **Oral care implementer:** □ Nurse □   Nurse's aide |
| --- | --- | --- |
|  | **C: Other interventions.** | |
|  | - 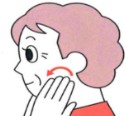Salivary gland massage (to increa production).   □ salivary gland massage 15 times   - Lip care: □ Apply lip balm daily | |
|  | **D: Prosthodontic care interventions.** | |
|  | - Use of individual denture marking kits - cleaned by nurses every night at 8 PM - physical cleaning with water (if necessary, use his own toothpaste and toothbrush to clean) - Filling the sink during cleaning to prevent the denture from falling out - Recording of daily cleaning and nighttime removal of the denture   Ultrasonic cleaning machine is available | |
| Effectiveness  evaluation | - Evaluation tool: Oral Health Assessment Tool (OHAT) - Evaluation time: after 3 months of drying period | |

se saliva

daily

**Daily record sheet for oral health promotion for the Older Persons in Geriatric Care Facilities (GCFs)**

Bed Number: Name: Entry Number: Charge Nurse: Nursing assistant:

**I. Oral health assessment:** (admission assessment, re-assessment on the 1st day of each month, see OHAT assessment form records for details)

**II Oral health promotion options:** □ brushing □ oral care □ denture care (can be multiple choice)

## Week 1 / Total 12 weeks

|  | Monday | Tuesday | Wednesday | Thursday | Friday | Saturday | Sunday |
| --- | --- | --- | --- | --- | --- | --- | --- |
|  | / | / | / | / | / | / | / |
| **After waking up in the**  **morning** | - Brushing teeth   □Oral Care | - Brushing teeth   □Oral Care | - Brushing teeth   □Oral Care | - Brushing teeth   □Oral Care | - Brushing teeth   □Oral Care | - Brushing teeth   □Oral Care | - Brushing teeth   □Oral Care |
| **Afternoon** | □Oral Care | □Oral Care | □Oral Care | □Oral Care | □Oral Care | □Oral Care | □Oral Care |

| **Before going to bed** | - Brushing   teeth   - Denture removal for cleaning and soaking | - Brushing   teeth   - Denture removal for cleaning and soaking | - Brushing   teeth   - Denture removal for cleaning and soaking | - Brushing   teeth   - Denture removal for cleaning and soaking | - Brushing   teeth   - Denture removal for cleaning and soaking | - Brushing   teeth   - Denture removal for cleaning and soaking | - Brushing   teeth   - Denture removal for cleaning and soaking |
| --- | --- | --- | --- | --- | --- | --- | --- |

Week 1 Special Situation Record: Nurse:

## Week 2 / Total 12 weeks

|  | Monday | Tuesday | Wednesday | Thursday | Friday | Saturday | Sunday |
| --- | --- | --- | --- | --- | --- | --- | --- |
|  | / | / | / | / | / | / | / |
| **After** | □ Brushing | □ Brushing | □ Brushing | □ Brushing | □ Brushing | □ Brushing | □ Brushing |
| **waking up** | teeth | teeth | teeth | teeth | teeth | teeth | teeth |
| **in the** | □Oral Care | □Oral Care | □Oral Care | □Oral Care | □Oral Care | □Oral Care | □Oral Care |
| **morning** |  |  |  |  |  |  |  |
| **Afternoon** | □Oral Care | □Oral Care | □Oral Care | □Oral Care | □Oral Care | □Oral Care | □Oral Care |
| **Before** | □ Brushing | □ Brushing | □ Brushing | □ Brushing | □ Brushing | □ Brushing | □ Brushing |
| **going to** | teeth | teeth | teeth | teeth | teeth | teeth | teeth |
| **bed** | □ Denture removal for cleaning and soaking | □ Denture removal for cleaning and soaking | □ Denture removal for cleaning and soaking | □ Denture removal for cleaning and soaking | □ Denture removal for cleaning and soaking | □ Denture removal for cleaning and soaking | □ Denture removal for cleaning and soaking |

Week 2 Special Situation Record: Nurse: _


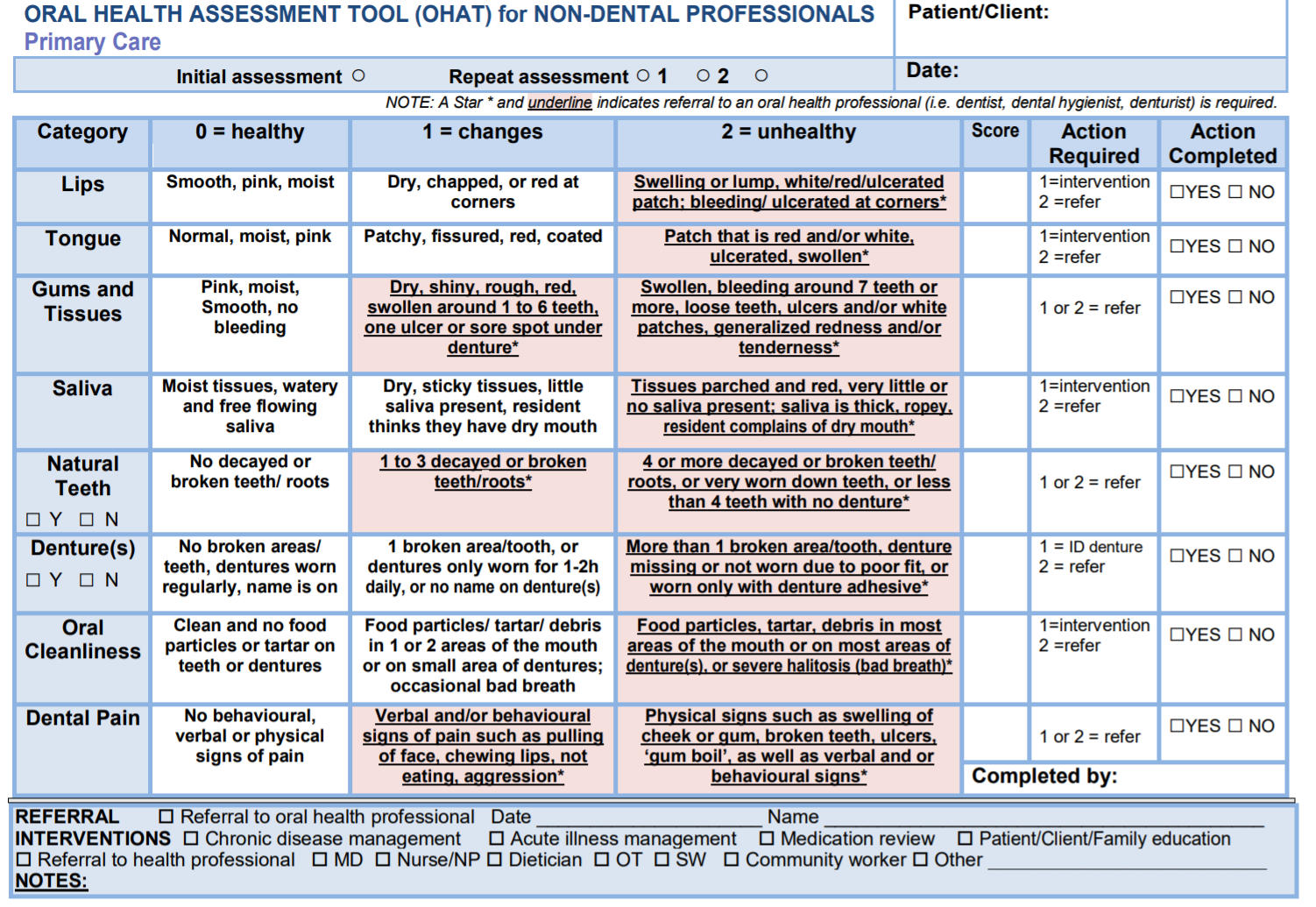


Self-Efficacy for Providing Mouth Care (SE-PMC) and Attitudes for Providing Mouth Care (A-PMC) Scale

| **items** | **1-strongly disagree** | **2-disagree** | **3-agree** | **4-strongly disagree** |
| --- | --- | --- | --- | --- |
| **Self-Efficacy for Providing Mouth Care: 11 items** |  |  |  |  |
| *Promoting Oral Hygiene* |  |  |  |  |
| I believe I can play a useful part in preventing my residents from getting gum disease. |  |  |  |  |
| I believe I can help in preventing my residents’ teeth from becoming decayed. |  |  |  |  |
| If I care for residents’ teeth correctly, their mouths will feel better. |  |  |  |  |
| If I brush and floss residents’ teeth correctly, I expect they will experience fewer dental problems. |  |  |  |  |
| I believe I can help independent residents have better mouth care. |  |  |  |  |
| *Providing Mouth Care* |  |  |  |  |
| I feel more uncomfortable brushing inside a resident’s mouth than I do with most other kinds of personal care.* |  |  |  |  |
| When I brush a resident’s teeth, I feel unsure if I am doing it right.* |  |  |  |  |
| I am not very good at providing mouth care to residents with dementia.* |  |  |  |  |
| *Obtaining Cooperation* |  |  |  |  |
| When a resident does not want me to brush his/her teeth, I can usually figure out a way to get the job done without forcing them. |  |  |  |  |
| I know ways to successfully provide mouth care to residents who hit or scream. |  |  |  |  |
| I can usually get my residents to cooperate with mouth cleaning. |  |  |  |  |
| **Attitudes for Providing Mouth Care: 11 items** |  |  |  |  |
| *Care of Residents’ Teeth* |  |  |  |  |
| Brushing teeth is a very personal thing that you should not be expected to do for somebody else.* |  |  |  |  |
| I think that the dentist is the only person who can help residents who have gum disease.* |  |  |  |  |
| If residents’ gums bleed, I feel I should probably stop brushing their teeth altogether.* |  |  |  |  |
| I think that only the dentist can prevent residents’ teeth from decaying.* |  |  |  |  |
| It is more important to take care of other needs of the residents instead of cleaning their mouths.* |  |  |  |  |
| In my opinion, it is better to wait until residents have a problem before asking the dentist to see them.* |  |  |  |  |
| *Care of Own Teeth* |  |  |  |  |
| It is important to me to keep all of my own teeth. |  |  |  |  |
| It is my own responsibility to look after the health of my mouth. |  |  |  |  |
| Up to now, I feel I have looked after my teeth well |  |  |  |  |
| If I was too ill or disabled to clean my own teeth, I hope somebody would do it for me. |  |  |  |  |
| I believe my own teeth should last me throughout my life. |  |  |  |  |

* means reverse coded.
